# The cytokine profile of women with severe anxiety and depression during pregnancy

**DOI:** 10.1186/s12888-019-2087-6

**Published:** 2019-04-03

**Authors:** P. Leff Gelman, I. Mancilla-Herrera, M. Flores-Ramos, M. F. Saravia Takashima, F. M. Cruz Coronel, C. Cruz Fuentes, A. Pérez Molina, J. Hernández-Ruiz, F. S. Silva-Aguilera, B. Farfan-Labonne, D. Chinchilla-Ochoa, S. Garza Morales, I. Camacho-Arroyo

**Affiliations:** 1 Instituto Nacional de Pernatologia, Isidro Espinosa de los Reyes, Montes Urales # 800, Col Lomas de Virreyes, 11000 (Mexico City), CD MX Mexico; 2 Instituto Nacional de Psiquiatría, 14370 CD MX, (Mexico City) Mexico; 3 Hospital General de México, Dr. Eduardo Liceaga, 06720 (Mexico City), CD MX Mexico; 4 HIPAM-Unidad de Investigación en Medicina-UNAM, Hospital General de México Dr. Eduardo Liceaga, 06720 (Mexico City), CD MX Mexico; 5 Unidad de Investigación en Reproducción Humana, Instituto Nacional de Perinatología-Facultad de Química, Universidad Nacional Autónoma de México, 04510 (Mexico City), CD MX Mexico; 6 Consejo Nacional de Ciencia y Tecnología/CONACyT, 03940 (Mexico City), CD MX Mexico

**Keywords:** Cytokines, Inflammation, Pregnancy, Depression, Anxiety

## Abstract

**Background:**

Controversial findings regarding the association between pro-inflammatory cytokines and depression have been reported in pregnant subjects. Scarce data about anxiety and its relationships with cytokines are available in pregnant women. To understand the association between anxiety and cytokines during pregnancy, we conducted the present study in women with or without depression.

**Methods:**

Women exhibiting severe depression (SD) and severe anxiety (SA) during the 3rd trimester of pregnancy (*n* = 139) and control subjects exhibiting neither depression nor anxiety (*n* = 40) were assessed through the Hamilton Depression Rating Scale (HDRS) and the Hamilton Anxiety Rating Scale (HARS). Serum cytokines were measured by a multiplex bead-based assay. Correlation tests were used to analyze the data and comparisons between groups were performed. A general linear model of analysis of variance was constructed using the group as a dependent variable, interleukin concentrations as independent variables, and HDRS/HARS scores and gestational weeks as covariables.

**Results:**

The highest levels of Th1- (IL-6, TNF-α, IL-2, IFN-γ), Th17- (IL-17A, IL-22), and Th2- (IL-9, IL-10, and IL-13) related cytokines were observed in women with SD + SA. The SA group showed higher concentrations of Th1- (IL-6, TNF-α, IL-2, IFN-γ) and Th2- (IL-4, and IL-10) related cytokines than the controls. Positive correlations were found between HDRS and IL-2, IL-6, and TNF-α in the SA group (*p* < 0.03), and between HDRS and Th1- (IL-2, IL-6, TNF-α), Th2- (IL-9, IL-10, IL-13) and Th17- (IL-17A) cytokines (*p* < 0.05) in the SD + SA group. After controlling the correlation analysis by gestational weeks, the correlations that remained significant were: HDRS and IL-2, IL-6, IL-9, and IL-17A in the SD + SA group (*p* < 0.03). HARS scores correlated with IL-17A in the SA group and with IL-17A, IL-17F, and IL-2 in the SD + SA group (*p* < 0.02). The linear model of analysis of variance showed that HDRS and HARS scores influenced cytokine concentrations; only IL-6 and TNF-α could be explained by the group.

**Conclusions:**

We found that the cytokine profiles differ when comparing pregnant subjects exhibiting SA with comorbid SD against those showing only SA without depression.

## Background

Major depressive disorder (MDD) is an important public health issue [1–3] that has been predicted to become the second leading cause of disability by the year 2020 (behind only ischemic heart disease) [4]. Compared to men, women are more than twice as susceptible to depression [5]. Depression during pregnancy is an important topic, both for understanding the pathophysiology of the disease and determining adequate treatment.

The prevalence of perinatal depression differs throughout pregnancy, from 7.4% in the first, 12.8% in the second, and 12.0% in the third trimester (20.4% considering the entire pregnancy) [5–7]. Nonetheless, other reports have shown that ~ 9% of women suffer major or minor depression during each of the trimesters, while postpartum depression has a prevalence of 13% (at 3 months postpartum) [8, 9]. Pregnant women experience more stressful events than non-pregnant women [10, 11], and frequently report tiredness or fatigue (87.2–96.5%) [12], symptoms commonly associated with depression and anxiety [13]. Interestingly, pregnant subjects with antenatal depression have a higher rate of preeclampsia and preterm birth associated with poor fetal and infant outcomes (motor and mental development) [10, 14].

It is well-known that Th1/Th2 and Th17/Treg immune balances are needed to maintain a successful pregnancy [5] and thereby of the conceptus [15]. Recent studies have shown that pregnant patients with MDD display a preferential inflammatory response [16, 17] and higher levels of circulating steroids (i.e., cortisol) when compared to healthy pregnant women [18].

Furthermore, human and animal studies have provided clear evidence that chronic activation of microglial cells [19–21] triggers mood disorders [22] through the release of a variety of neurotoxins, pro-inflammatory cytokines, free radicals, nitric oxide, chemokines, proteinases, and eicosanoids [19], causing neuronal dysfunction and/or aggravating underlying pathologies [23].

Several pieces of evidence support the premise that chronic psychological distress contributes to adverse pregnancy outcome, such as preterm birth, proposing that complex interactions among stress, individual health behaviors, genomic background, and the physiology of pregnancy itself might determine both maternal and child outcomes [24–26]. Such interactions involve immune mediators, such as interleukins (IL)-1β, IL-6, IL-10, TNF-α, hormones, and several other compounds (i.e., prostaglandins, chemokines) [5, 27].

With regard to mood-related disorders during pregnancy, multiple studies shed controversial results between depressive scores (CES-D, EPDS) and blood levels of Th1- and Th2-related biomarkers in women exhibiting MDD during mid and late pregnancy and the early postpartum period [28–30]. In line with this, one study found positive correlations between depressive scores and circulating levels of IL-6 and TNF-α [28]; whereas other studies have reported non-significant associations between Th1-related (IL)-6) and Th2-related (IL)-10) biomarkers and depressive symptoms during pregnancy [29]. However, this latter study reported that depressive symptoms during pregnancy, as well as the IL6 and IL-10 biomarkers, were significant predictors of postpartum Edinburgh Perinatal Depression Scale (EPDS) score [29].

A similar study focused on exploring the relationship between prenatal psychological symptoms, prenatal stress, and the increased risk for atopic diseases in the offspring found positive correlations between concentrations of potentially proallergenic cytokines (IL-5, IL-13) and maternal prenatal psychological symptoms [30]. This study also showed that a higher (IFN)-γ/IL-4 ratio positively correlated with the depressive score; whereas IL-12 and IL-13 correlated with pregnancy-related anxiety score; besides, IL-9 and IL-13 displayed significant correlations with mood-related symptoms in women exhibiting depression and anxiety from early to mid-gestation [30].

An interesting study demonstrated positive correlations between Th1 (IL-6) and Th2 (IL-10) cytokines with depression, anxiety, and perceived stress in women during mid-pregnancy [14–28 gestation weeks (gwks)]. Moreover, the cited study showed that plasma levels of IL1-β, IL-6, and IL-10 were significantly lower among women who took selective serotonin reuptake inhibitors (SSRI) during pregnancy [27]. This study provides evidence that major depression and severe anxiety during pregnancy significantly contribute to immune system pathways, thereby enhancing the risk of a poor pregnancy with negative obstetric outcomes, as previously reported [5].

Despite a large number of studies exploring the associations between depression and inflammation [31], little is known about the relationship between anxiety and inflammation during pregnancy [32]. Recent studies have shown that several physiological/biochemical variables are associated with prenatal anxiety, such as polyunsaturated fatty acids, elevated cortisol, low levels of the enzyme that metabolizes cortisol, and elevated pro-inflammatory cytokines (IL1, IL-6, TNF-α) [33]. Thus, despite the associations between inflammatory biomarkers and depressive disorders during pregnancy, the link between immune mediators and anxiety remains unclear.

In such a context, we formulated the following hypothesis: a) pregnant women exhibiting severe anxiety with or without comorbid depression should display a significant increase in distinct immune mediators in blood when compared to healthy pregnant women; b) the increase in Th1 and/or Th2-immune mediators should display significant correlations with anxiety and depressive symptoms, reflecting the influence of affective disorders in the dysregulation of the maternal immune system and immune balance (Th1:Th2) in pregnancy.

## Methods

### Design of the Study

We conducted a transversal study at the National Institute of Perinatology (Neuroscience Department, Mexico City) together with the Department of Gynecology and Obstetrics (GO) at the General Hospital of Mexico (HGM, Dr. Eduardo Liceaga, Mexico City) from October 2014 to December 2016. Approval from the Institution Ethical Committee was obtained before the beginning of the study (HGM, D1/14/112/04/072, 2014–2016). Written informed consent was obtained from all women recruited before the study began. Pregnant women were admitted at the Prenatal Control Outpatient Unit of the GO Department during the second and third trimester of gestation. Clinical, sociodemographic, obstetric, and psychiatric characteristics of the recruited participants were recorded into a database. At entry, participants in the study required first, second, and third-trimester lab tests (blood count, biochemical testing, urinalysis, thyroid function), 2D fetal ultrasound, and Doppler Monitoring, including lab test for sex hormones, cortisol, and DHEA-S (data not shown). All patients were inhabitants of Mexico City and surrounding areas.

### Participants

Patients recruited to the study attended the Prenatal Control Outpatient Unit. Women between 16- and 30-years-old, exhibiting a pregnancy between 20 and 39 gwks, were invited to participate in the study. In their first visit, a complete clinical evaluation was carried out, including anthropometric evaluation. Participants were asked to complete the self-reported questionnaires used to measure anxiety and depressive symptoms; in the same interview, an evaluation of socio-demographic variables was done, including marital status, education level, and working status. Patients exhibiting a cutoff score > 15 in the Hamilton Anxiety rating scale (HARS) were considered as having severe anxiety. Patients with a cutoff score as < 5 were included in the control group. We excluded patients who were receiving psychotropic medication, patients with illicit substance use, previous psychiatric diagnosis, obstetric pathologies (diabetes, hypertension, preeclampsia), infections, and medical diseases (past and present neurological, metabolic, cardiovascular, degenerative, and rheumatic disorders). Upon completion of the questionnaires, participants displaying high-rating scores for moderate to severe anxiety and/or depressive symptoms were remitted to a psychiatric outpatient service for mood-disorder management. After psychiatry clinical interview and completion of the questionnaires, patients were remitted to the GO Department for blood extraction and analysis of their serum cytokine profile.

We assessed a total of 298 pregnant women: patients, exhibiting low to high levels of depressive symptoms and comorbid anxiety (*n* = 141), pregnant women exhibiting anxiety symptoms without depression (*n* = 100) mostly during the 3rd trimester of pregnancy (28–40 gwks), and healthy pregnant women displaying no affective disorders (*n* = 57). All participants were screened for depressive symptoms and anxiety, serum cytokines and sociodemographic parameters. Some of the participants were excluded due to incomplete questionnaires, absence or incomplete laboratory tests, or inconsistencies in the evaluation of both depressive and anxiety symptoms. Our present work included three groups of participants a) patients exhibiting severe anxiety without depression (SA, *n* = 72); b) patients exhibiting severe anxiety and comorbid severe depression (SA + SD, *n* = 67); and c) healthy pregnant women as controls (CTRL, *n* = 40).

### Criteria and evaluation instruments

Criteria considered for patient recruitment included the presence of severe anxiety according to the 14 item-Hamilton Anxiety Rating Scale (HARS) [34], which assesses the severity of anxiety symptoms with a cutoff score > 15 for severe anxiety. Depressive symptoms were evaluated using the 17-Item Hamilton Depression Rating Scale (HDRS), with a cutoff score > 19 for major depressive disorder [35, 36]; and evaluated by psychiatric clinical interview following the Diagnostic and Statistical Manual of Mental Disorders (DSM-5; American Psychiatric Association, 2015). HDRS and HARS were applied to healthy women to confirm the absence of depressive and anxiety symptoms.

Both instruments have been validated in the local language [36, 37] and have been demonstrated to be reliable, specific, and sensitive [38]. Rating scores obtained from each instrument were checked by the Psychiatric Department, and final evaluations were recorded in a clinical database. Patients exhibiting an HDRS score > 19 and HARS score > 17 were referred to the psychiatry department for further evaluation and treatment.

### Blood sampling

Blood sampling was carried out during daylight under aseptic conditions, and 5.0 mL of venous blood was collected into sterile 13 × 100/Vacutainer BD Hemogard Tubes, containing Clot Activator/Polymer Gel for serum separation (Becton & Dickinson 367,977, USA). Tubes were allowed to clot for 1 h at 4 °C before serum separation. Fresh serum was collected by centrifuging samples at 1600 × G for 10 min, aliquoted into 1.5-mL Eppendorf tubes. and stored at − 70 °C until further use.

### Quantification of serum cytokine profile

Serum cytokines (IFN-γ, TNF-α, IL-6, IL-2, IL-5, IL-13, IL-4, IL-10, IL-9, IL-17A, IL-17F, IL-21 and IL-22) were determined by an immunoassay based on multiplex bead array using the LEGENDplex™ Human Th Cytokine Panel (Cat. 740,001, BioLegend, USA) following the manufacturer’s protocol. Briefly, serum samples (50 μL) were incubated with specific IL-antibody coated beads in a mix buffer at room temperature (RT) for 1.5 h. An antigen-antibody reaction was carried out at RT for 1.5 h in the presence of specific fluorochrome per cytokine. After washes, samples were analyzed in a FACS Aria III flow cytometer (BD, USA). The concentration of analytes was calculated using the LEGENDplexTM Data Analysis Software v 7.0 (Biolegend, CA, USA). The limit of detection (LD) values detected for Th1 cytokines (IL-6, TNF-α, IL-2, IFN-γ) were 12.63 pg/ml, 78.13 pg/ml, and 9.88 pg/ml, respectively.

The LD values of Th17-related (IL-17A, IL-21, and IL-22) cytokines were 78.13 pg/ml, whereas the LD value of IL-17F was 14.1 pg/ml respectively; the LD values detected for Th2 cytokines (IL-4, IL-5, IL-13, IL-10, IL-9) were 78.13 pg/ml, 9.25 pg/ml, 11.68 pg/ml, 88.97 pg/ml and 6.77 pg/ml respectively.

### Statistical analysis

Cytokine concentrations were presented as mean ± SEM. Demographic parameters were expressed as mean ± SD. Bivariate analyses, as well as parametric and non-parametric t-tests, were conducted to assess the associations among immune mediators, demographic parameters, and psychological distress scores. The non-parametric t-test analysis with Welch’s correction was used to detect significant differences between the demographic measures and groups, as well as Th-related cytokines among the tested groups. Partial correlation analysis was used to detect significant differences between serum cytokines and demographic parameters, after controlling specific parameters in the studied groups. A post hoc Tukey test analysis was performed to estimate the differences between cytokines and demographic parameters among groups. Also, a general linear model of analysis of variance was constructed using the group as a dependent variable, interleukin concentrations as independent variables, and HDRS/HARS scores and GWKS were introduced as covariables. Statistical analyses were performed using GraphPad Prism 7 (GraphPad Softwares Inc. USA) and SPSS software v.24.0 (Armonk, NY: IBM Corp). For all the statistical analysis, the *p*-value < 0.05 was considered significant.

## Results

### Demographic characteristics

Table 1 shows the sociodemographic characteristics of participants (*n* = 179). Participants were predominantly Latin women in the 3rd trimester of pregnancy with a gestation week average of 36.4 (range, 20.5–41.1 gwks) and an average age of 26.2 (range 16–39 years).

**Table 1:**
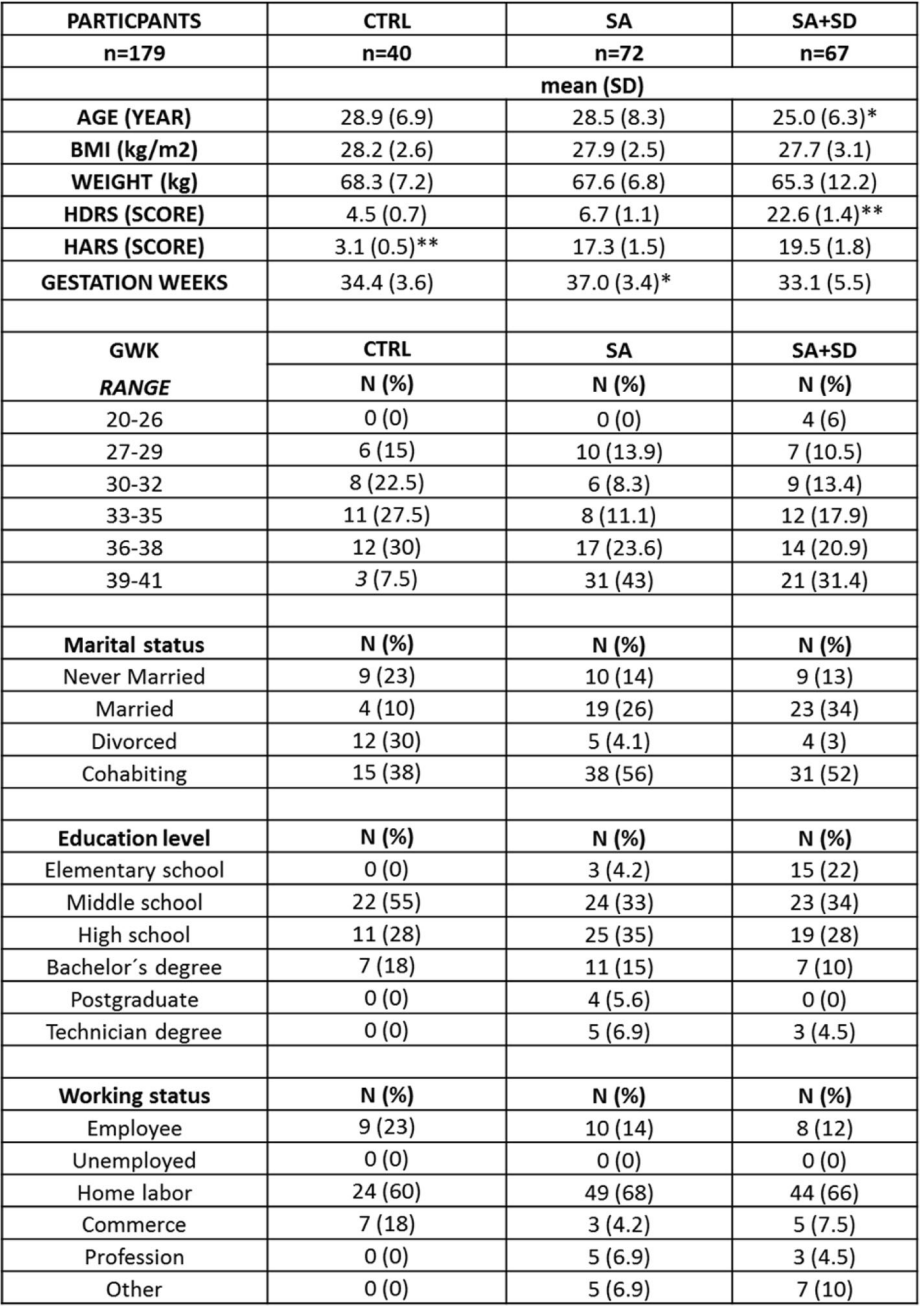
The non-parametric, t-test with Welch’s correction was used to detect statistical differences between demographic measures among the studied groups

(*) *p* < 0.005, indicates the differences in age and gwks found among tested groups. (**) *p* < 0.001, indicates the differences found between the final HDRS score values estimated for either the SA + SD and the CTRL groups, respectively; or between the final HARS score values estimated for each of the studied groups. Data are expressed as the mean ± SD. Data were calculated using GraphPad-v.7

Abbreviations: *SA + SD* severe anxiety plus comorbid severe depression; *SA* severe anxiety, *CTRL* control, *HDRS* Hamilton Depression Rating Scale, *HARS* Hamilton Anxiety Rating Scale, *BMI* Body Mass Index, *GWK* gestational weeks

#### Serum cytokine concentrations

**Fig. 1:**
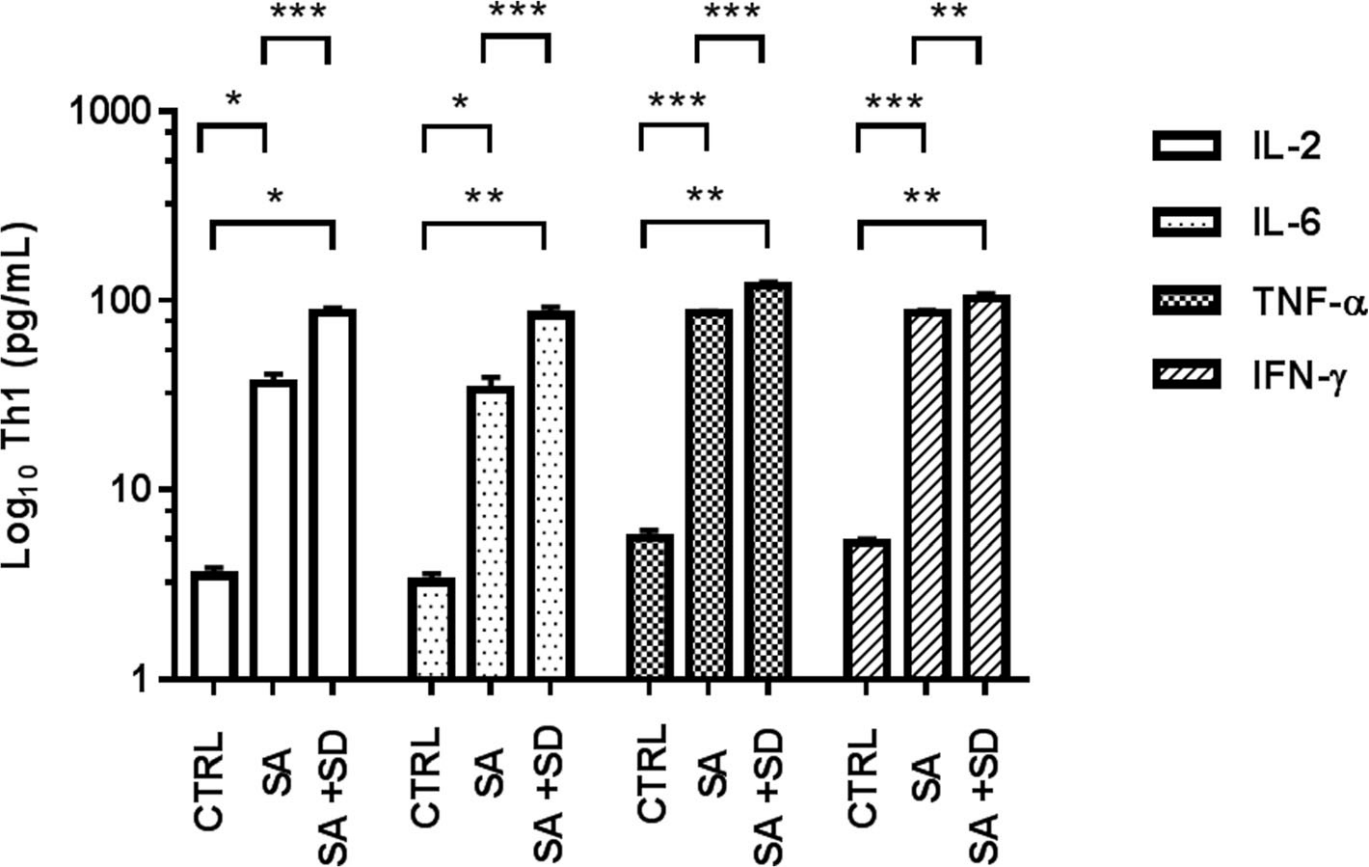
Concentrations of Th1-related cytokines in tested groups. The figure depicts the Log_10_ concentration values of estimated Th1-related cytokines in serum in the CTRL, SA, and SA + SD groups, respectively. Th1-related cytokines were quantified by flow cytometry (see methods). The concentration values of proinflammatory cytokines are expressed as the mean ± SEM, as described: CTRL; IL-2 (3.5 ± 0.4 pg/mL), IL-6 (3.3 ± 0.4 pg/mL), TNF-α (5.6 ± 0.5 pg/mL), IFN-γ (5.3 ± 0.3 pg/mL). SA; IL-2 (33.7 ± 5.7 pg/mL), IL-6 (33.3 ± 5.9 pg/mL), TNF-α (85.9 ± 1.8 pg/mL), IFN-γ (86.5 ± 2.1 pg/mL). SA + SD; IL-2 (85.8 ± 5.7 pg/mL), IL-6 (83.4 ± 9.2 pg/mL), TNF-α (119.5 ± 6.2 pg/mL), IFN-γ (103.5 ± 5.4 pg/mL). The non-parametric, t-test analysis with Welch’s correction was used to estimate the p-values for each Th2-related cytokine assayed among the studied groups. (*) *p* ≤ 0.05; (**); *p* ≤ 0.001; (***) *p* ≤ 0.0001. Significant differences were established at a *p* < 0.05. Abbreviations: SA + SD, severe anxiety plus comorbid severe, severe anxiety; CTRL; control. Data were calculated using GraphPad v.7

**Fig. 2:**
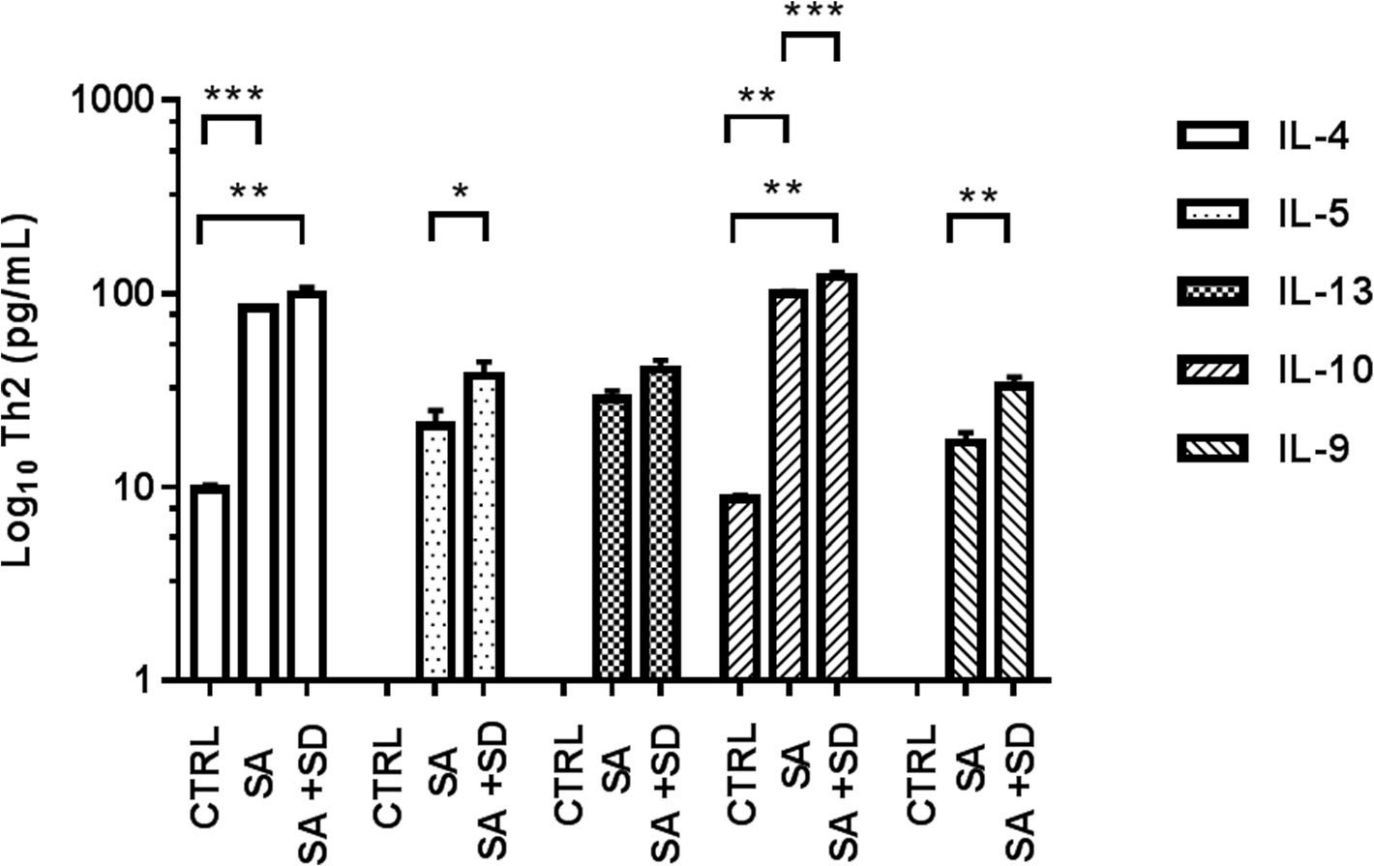
Concentrations of Th2-related cytokines in the tested groups. The figure depicts the Log_10_ concentration values of estimated Th2-related cytokines in serum in the CTRL, SA, and SA + SD groups, respectively. Th2-related cytokines were quantified by flow cytometry (see methods). The concentration values of anti-inflammatory cytokines are expressed as the mean ± SEM, as described: CTRL; IL-4 (9.7 ± 0.5 pg/mL), IL-5, ND; IL-13, ND; IL-10 (8.7 ± 0.4 pg/mL), IL-9, ND. SA; IL-4 (84.3 ± 2.0 pg/mL), IL-5 (20.7 ± 4.2 pg/mL), IL-13 (28.6 ± 2.9 pg/mL), IL-10 (99.8 ± 2.7 pg/mL), L-9 (16.9 ± 2.2 pg/mL). SA + SD; IL-4 (99.1 ± 8.2 pg/mL), IL-5 (37.5 ± 6.5 pg/mL), IL-13 (40.3 ± 4.9 pg/mL), IL-10 (121.4 ± 7.9 pg/mL), IL-9 (33.2 ± 3.7 pg/mL). The non-parametric, t-test analysis with Welch’s correction was used to estimate the *p* values for each Th2-related cytokine assayed among the studied groups. (★) non-determined values; (*) *p* ≤ 0.05; (**); *p* ≤ 0.001; (***) *p* ≤ 0.0001. Significant differences were established at a *p* < 0.05. Abbreviations: ND, non-determined; SA + SD, severe anxiety plus comorbid severe depression; SA, severe anxiety; CTRL; control. Data was calculated using GraphPad v.7

**Fig. 3:**
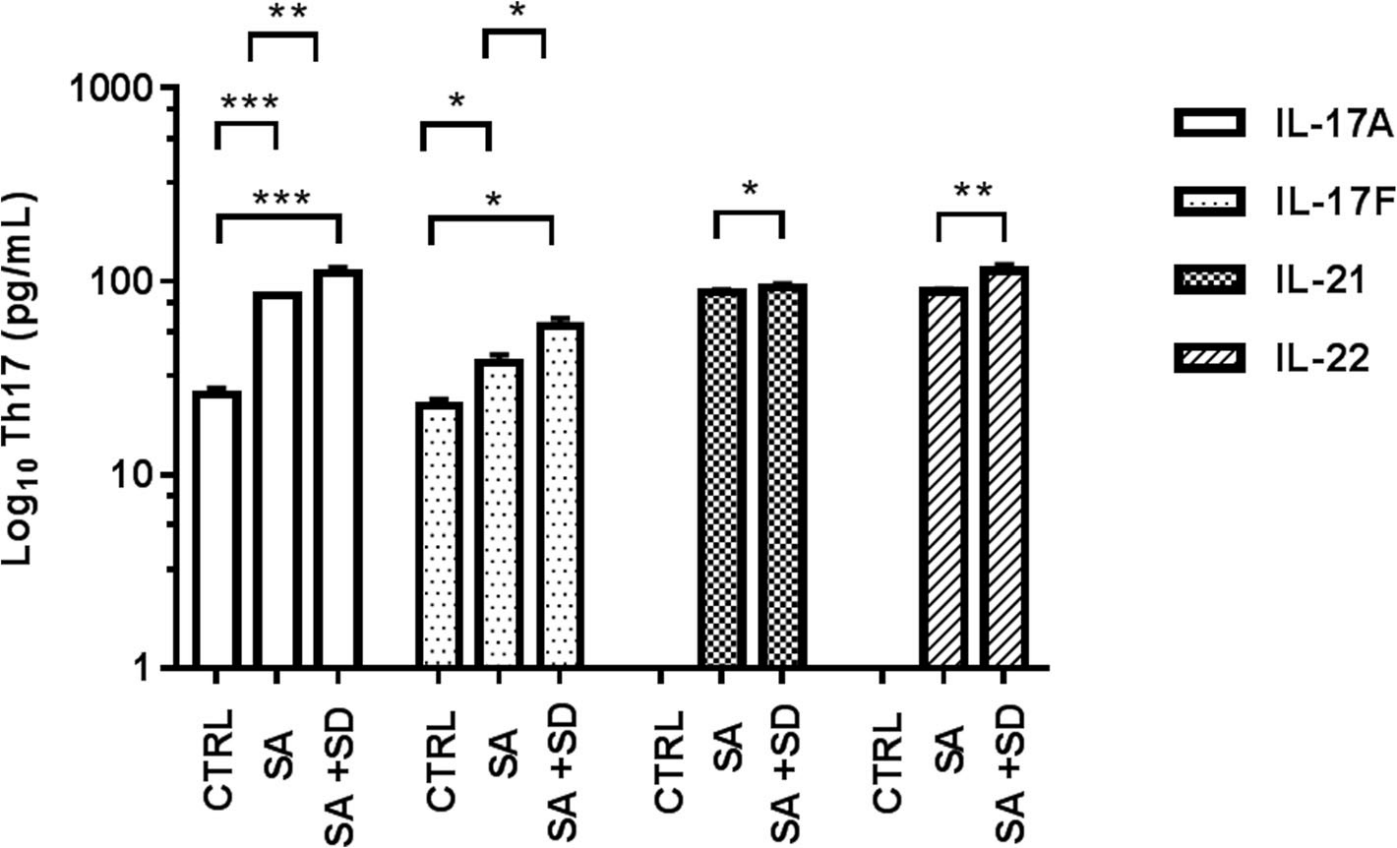
Concentrations of Th17-related cytokines in tested groups. The figure depicts the Log_10_ concentration values of estimated Th17-related cytokines in serum in the CTRL, SA, and SA + SD groups, respectively. Th17-related cytokines were quantified by flow cytometry (see methods). The concentration values of proinflammatory Th17-related cytokines are expressed as the mean ± SEM, as described: CTRL; IL-17A (25.9 ± 2.1 pg/mL); IL-17F (22.7 ± 1.8 pg/mL); IL-21, ND; IL-22, ND. SA; IL-17A (84.6 ± 1.3 pg/mL), IL-17F (38.1 ± 3.7 pg/mL), IL-21 (87.9 ± 2.4 pg/mL), IL-22 (88.9 ± 2.7 pg/mL). SA + SD; IL-17A (109. 9 ± 1 8.3 pg/mL), IL-17F (59.1 ± 5.5 pg/mL), IL-21 (92.6 ± 4.5 pg/mL), IL-22 (113.5 ± 8.6 pg/mL). The non-parametric, t-test analysis with Welch’s correction was used to estimate the p values for each Th2-related cytokine assayed among the studied groups. (★) non-determined values; (*) *p* ≤ 0.05; (**); *p* ≤ 0.001; (***) *p* ≤ 0.0001. Significant differences were established at a *p* < 0.05. Abbreviations: ND, non-determined; SA + SD, severe anxiety plus comorbid severe depression; SA, severe anxiety; CTRL; control. Data were calculated using GraphPad v.7

Figures 1, 2, 3 depict the Log_10_ serum concentrations of Th1, Th2, and Th17-related cytokines in the studied groups. As shown, higher concentrations of proinflammatory cytokines (IL-2, IL-6, TNF-α, IFN-γ) were observed in the SA + SD group, as compared with the values detected in the SA and the CTRL groups (Fig. 1).

Similarly, higher concentrations of anti-inflammatory (Th2)-related cytokines (IL-4, IL-5, IL-13, IL-10, IL-9) were observed in the SA + SD group, as compared with the values found either in the SA or the CTRL group (Fig. 2).

Furthermore, higher concentrations of (Th17)-related cytokines (IL-17A, IL-17F, IL-21, IL-22) were found in the SA + SD group, when compared to the values displayed by the SA group, or between the IL-17A and IL-17F concentrations and those observed in the CTRL group. The highest levels of IL-17A and IL-22 were detected in SA + SD (Fig. 3).

#### Correlations between serum cytokine concentrations and demographic measures

Table 2 describes the correlations found between the serum cytokine profile and demographic parameters. As shown, the SA + SD group showed more correlations between cytokines concentrations and studied variables when compared to the SA and CTRL groups. The SA + SD displayed positive correlations between Th1-related (IL)-2, (IL)-6, and TNF-α cytokines and the depression score (HDRS), including the Th2-related (IL)-13, (IL)-10, and (IL)-9 mediators and Th17-associated (IL)-17A cytokines, respectively. The SA group showed similar positive correlations between Th1-related (IL)-2, (IL)-6, and TNF-α cytokines and the HDRS score. Note, however, that only IFN-γ correlated either positively or negatively with the anxiety (HARS) score in the SA + SD and the CTRL groups, respectively. Significant differences were observed between cytokines and both HDRS (Tukey test, *p* ≤ 0.05) and HARS scores (Tukey test, *p* ≤ 0.03) in both SA + SD and the SA groups, respectively (data not shown).

**Table 2:**
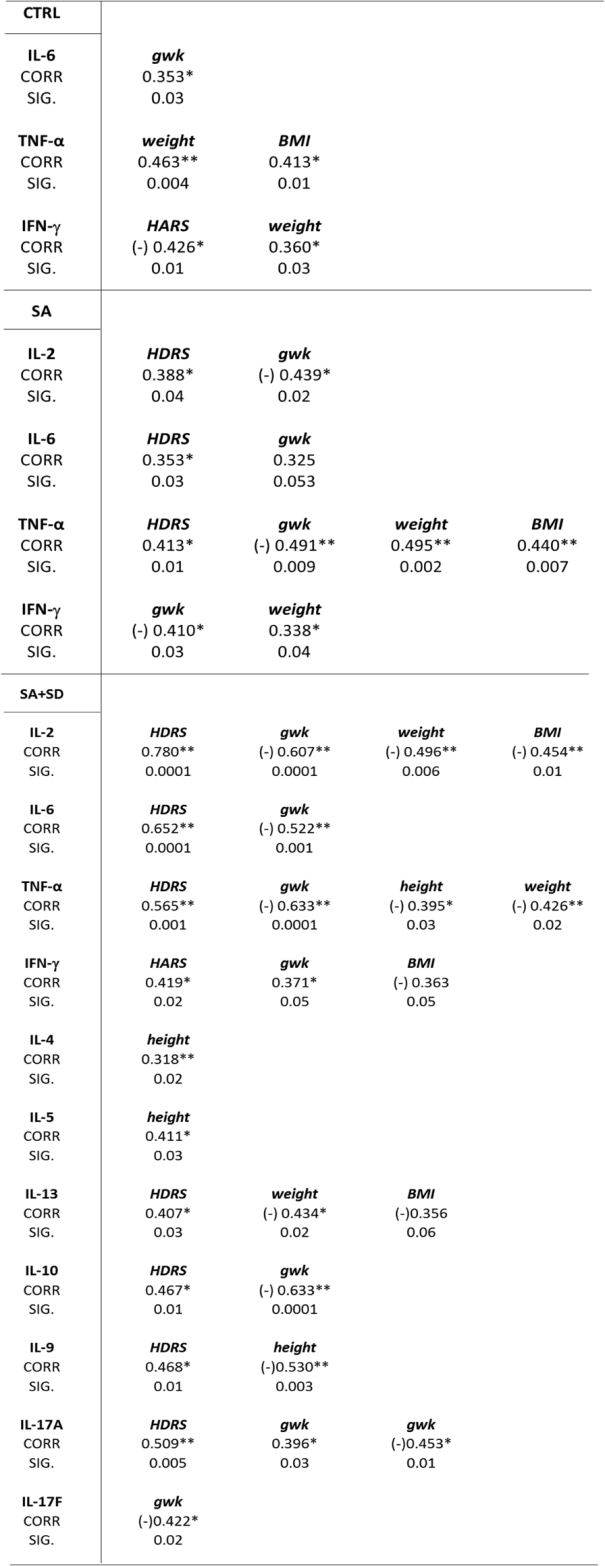
SSPS software v.24.0 was used to determine the Pearson-bilateral correlations among tested groups

Abbreviations: *SA + SD* severe anxiety plus comorbid severe depression, *SA* severe anxiety, *CTRL* control, *HDRS* Hamilton Depression Rating Scale, *HARS* Hamilton Anxiety Rating Scale, *BMI* Body Mass Index, *GWK* gestational weeks, *Corr* correlation, *Sig* significance. (*) Significant correlation at a *p*-value of 0.05; (**) Significant correlation at a *p*-value of 0.01

#### Correlations between cytokine concentrations and age, anxiety and depressive scores adjusted by gestation weeks

Table 3 depicts the correlations between the Th-related cytokines concentration and demographic measures when controlling by gestation weeks. As shown, after controlling for this variable, the independent Th-related variables, such as Th1-(IL-2, IL-6), Th2-(IL-9), and Th17-(17A, 17F)-related cytokines positively correlated with both HDRS and HARS scores in both the SA + SD and SA groups, respectively. Furthermore, negative correlations were found between age and Th-related cytokines. Significant differences were observed between the cytokines assayed and HARS (Tukey test, *p* ≤ 0.02) and HDRS scores (Tukey test, p ≤ 0.03) in both the SA + SD and SA groups, respectively (data not shown).

**Table 3:**
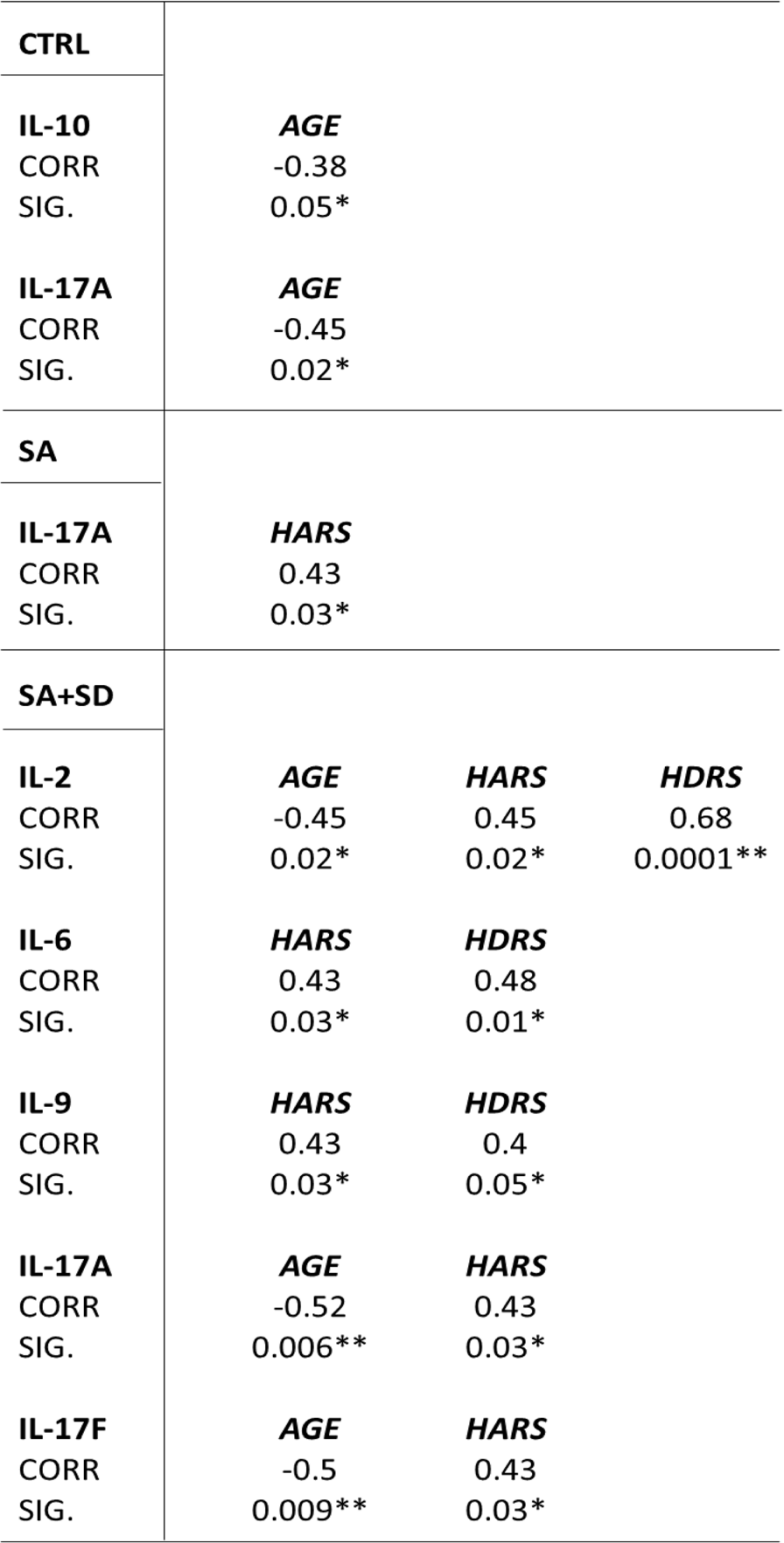
SSPS software v.24.0 was used to determine the Pearson correlations between parameters among tested groups

Correlations and *p*-values were obtained after controlling gestational weeks, used as the dependent variable for the analysis (see text for details)

Abbreviations: *SA + SD* severe anxiety plus comorbid severe depression, *SA* severe anxiety, *CTRL* control, *HDRS* Hamilton Depression Rating Scale, *HARS* Hamilton Anxiety Rating Scale, *GWK* gestational weeks, *Corr* correlation, *Sig* significance. (*) Significant correlations at a p-value ≤0.05; (**) Significant correlation at a p-value ≤0.01

**Table 4:**
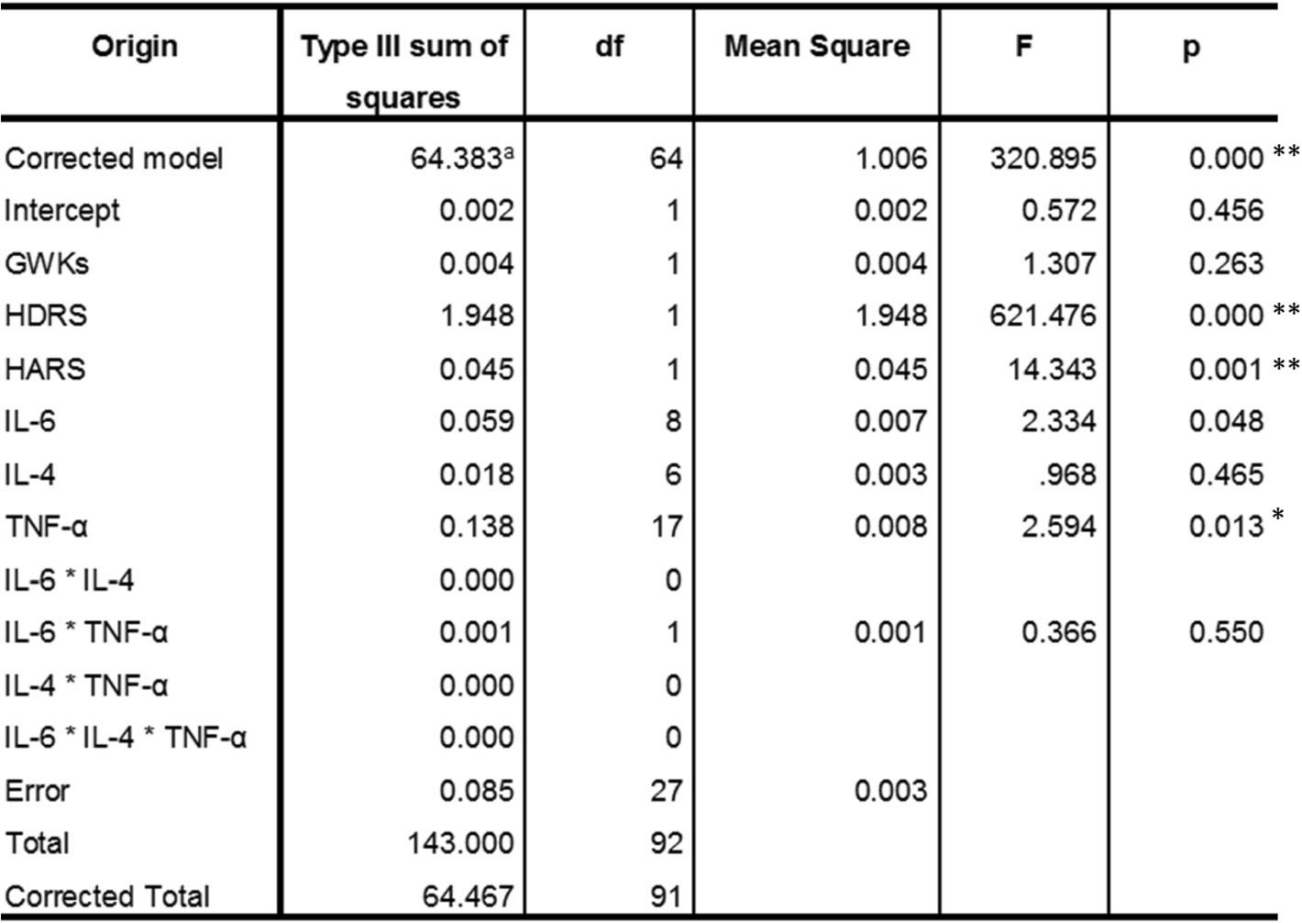
Test between subject and effects

Dependent variable: groups CTRL, SA, SA + SD. (a) R-squared = 0.965 (Adjusted R-squared = 0.996)

Abbreviations: *HDRS* Hamilton Depression Rating Scale, *HARS* Hamilton Anxiety Rating Scale, *GWKs* gestational weeks. Significant differences were established at a *p*-value ≤0.05. (*) Significant differences at a *p*-value ≤0.05; (**) Significant differences at a *p*-value ≤0.01

#### Th1:Th2 ratio

Th1:Th2 ratios were estimated in each of the tested group. The estimated Th1:Th2 ratios (IFN-γ,-IL-4 and TNF-α-IL4 ratios) in the control group were 0.5 and 0.6 respectively; whereas the Th1 (IFN-γ,TNF-α):Th2(IL-4) ratios estimated in the SA + SD group were 1.3 and 1.4, respectively. The estimated Th1:Th2 ratio values in the SA group were 0.9 for both the IFN-γ:IL-4 and TNF-α:IL-4 ratios.

#### General linear model

A general linear model was constructed to assess the relationship between biological and sociodemographic variables (Table 4). We used all studied groups as the dependent variable and serum concentrations of cytokines as the independent variable. Both HDRS and HARS scores were included as covariables. The model showed that both HDRS and HARS were significantly associated with interleukin concentrations among the studied groups; whereas only IL-6 and TNF-α concentrations were defined by the groups. The model explained 96.5% of the variance, and the model provided a good fit of the data (R-squared = 0.965; adjusted R-Squared = 0.996).

## Discussion

Our study comprised pregnant participants (*n* = 179) who were mostly recruited during the 3rd trimester of pregnancy. Cytokine concentrations varied as pregnancy progresses. Pro-inflammatory cytokines tend to increase at the final weeks of pregnancy, while anti-inflammatory cytokines show an opposite profile [39]. Some authors have argued that Th1-related cytokines play a crucial role in subjects exhibiting both depression and anxiety during pregnancy. In line with this, several studies have extensively documented that psychosocial stress and depressive symptoms are associated with elevations of inflammatory biomarkers in non-pregnant humans and animals [40, 41]. In a similar context, perceived stress with elevated stress scores positively correlate with high IL-6 and TNF-γ serum levels and with low IL-10 levels in healthy subjects exhibiting normal pregnancies [42].

Similar studies conducted in pregnant women have shown significant correlations between depressive symptoms and Th1/Th2-related biomarkers (IL-6, TNF-α, IL-10) at mid and late pregnancy [17, 29, 42]; in addition to the increased plasma levels of IL-1β and IFN-γ, prenatal stress, and anxiety symptoms observed in pregnant women exhibiting high levels of anxiety after undergoing elective cesarean [43].

Recent findings show that anxiety is associated with increased levels of interleukin-6 (IL-6) and associated with an increased risk for diseases with an inflammatory etiology [44]. In a similar context, anti-inflammatory cytokines could play distinct roles in mood-associated disorders, as suggested in recent studies that show a correlation between Th2-related IL-13/IL-9 cytokine concentrations and the depressive Edinburgh Postnatal Depression Scale (EPDS) score; whereas IL-13 and IL-10 cytokine concentrations correlated with anxiety symptoms in pregnant women during mid-pregnancy [30].

Interestingly, the levels of proinflammatory cytokines were higher in women exhibiting anxiety and comorbid depression with respect to pregnant women displaying only anxiety. Nonetheless, some studies showed opposite results, as shown for a single case study that reported that pro-inflammatory cytokine levels were unrelated with depression and anxiety symptoms [28].

Some authors have reported a correlation between higher levels of IL-6 and TNF-α and depression scores (CES-D) in subjects displaying major depression, but not with high scores for stress perception in Afro-American and Caucasian pregnant women [29]. Our results suggest that variations in cytokine concentrations are likely influenced by the intensity of depressive symptoms. We show that pregnant women exhibiting severe anxiety and comorbid depression displayed significant increases in serum levels of Th1 and Th17-related cytokines, which correlated with HDRS scores.

Furthermore, a recent study conducted by Osborne [45] showed that women exhibiting high levels of depressive and anxious symptoms in the third trimester of pregnancy showed a proinflammatory burst of Th1-related cytokines. Interestingly, these authors assessed serum cytokines at five points during pregnancy and observed differences over time [45].

Such observations led us to control our correlation analysis by gestational weeks. As shown, only IL-2, IL-6, IL-9, and IL-17A cytokines remained as relevant immune mediators that significantly correlated with the HDRS score in the SA + SD group. Such observations suggest that regardless of the gestational week period, depressive symptoms appear to modify the cytokine profile, showing that higher levels of depressive symptoms are related to increased levels of proinflammatory cytokines. Moreover, the observed increases in both IFN-γ: IL-4 and TNF-α: IL-4 ratios in the SA and SA + SD groups suggest that an inflammatory process takes place in vulnerable subjects with severe anxiety, becoming conspicuous as depression emerges in women displaying severe depression.

Interestingly, using the general linear model of analysis of variance constructed to explain the interaction between variables among the studied groups, we observed that the rating scores obtained from both depression (HDRS) and anxiety (HARS) instruments applied to the participants were associated with interleukins’ concentrations. However, only IL-6 and TNF-α cytokines were identified as relevant pro-inflammatory biomarkers that could differentiate between the groups exhibiting affective symptoms (depressive versus the anxiety/non-depressive group) and the control group.

Both IL-2 and IL-6 are well recognized as pro-inflammatory cytokines, while IL-9 is considered a pleiotropic cytokine that has direct and indirect effects on multiple cell types that affect immunity and inflammation [46].

Based on the high degree of plasticity and differentiation of Th17 cell lines into Th1 or Treg cells [47], it might be feasible to posit that such Th17-related cytokines could elicit qualitatively distinct responses and interactions with the brain and placenta, promoting the expression of depressive and anxiety symptoms in vulnerable women at high risk of developing mood disorders [5].

Finally, the activation of specific cytokine-associated signaling pathways in the brain and placenta has been proposed as an immune-related mechanism that appears to play a crucial role in triggering and/or exacerbating depressive symptoms during pregnancy, enhancing negative perinatal outcomes [5, 16–18, 24–26].

### Conclusions and perspectives

Our data show that patients exhibiting severe anxiety and comorbid depression display significant increases in serum proinflammatory and anti-inflammatory-related cytokines that correlated with the intensity of depressive symptoms measured by HDRS. Furthermore, the present results support much of interacting network systems driven by the immune and neuroendocrine systems and placenta [5–8, 18, 19], and whose dysregulation might precipitate or exacerbate depressive symptoms in vulnerable women exhibiting severe anxiety during pregnancy.

Our results parallel other studies that have shown that Th1 cytokines appear to modulate anxious symptoms in non-pregnant populations with increased levels of Th1 cytokines and higher ratios in the Th1:Th2 immune balance [48]. Most of our correlations found between depression and anxiety scores with Th(1), Th(2), and Th(17)-related cytokines support the role of inflammatory cytokines in mood-associated disorders during pregnancy.

As anxiety and depression are usually comorbid, it is imperative to perform studies to explore further correlations between biological mediators and anxiety symptoms in women at high risk of developing severe depression during mid to late pregnancy. It is worth noting that the emergence of depression associated with an immune-related inflammatory process and expression of a Th1-cytokine profile during pregnancy should be taken seriously. Activation of the innate immune system and their immune mediators might lead to a chronic inflammatory process that, if not controlled, might precipitate in both negative obstetric and neonatal outcomes [5]. Thus, our results show that depressive symptoms, regardless of anxiety and gestational weeks, are highly related to the increase of blood proinflammatory cytokines.

### Limitations

Several limitations in the present study should be noted. First, we applied the self-reported HARS and HDRS instruments used to evaluate depressive and anxiety symptoms, which have not been extensively used in pregnant women, as compared to other psychological instruments (e.g., the CES-D, EPDS, STAI, and GAD-7). Second, blood sampling was performed at a single time (T1) at the entry of participants into the study. Thus, to detect changes in cytokine concentration during late pregnancy two specific blood-sampling time-points (T1, T2) must have been elected as the optimum (i.e., at 28 and 39 gwk) in our studied population. Third, we did not evaluate the prevalence of cigarette smoking in recruited participants, an important variable that might have influenced our results. Also, pre-gestational weight could contribute to further information about the relevance of weight gain and BMI associated with the determination of a Th1, Th2 cytokine profile.

## Abbreviations

CES-D: Center for Epidemiological Studies Depression Inventory
CTRL: control
DHEA-S: Dehydroepiandrosterone sulfate
DSM-5: Diagnostic and Statistical Manual of Mental Disorders
EPDS: Edinburgh Perinatal Depression Scale
GAD-7: Generalized Anxiety Disorder Screener
GWK: gestation weeks
HARS: Hamilton Anxiety Rating Scale
HDRS: Hamilton Depression Rating Scale
IFN: interferon
IL: interleukins
MDD: major depressive disorder
SA: severe anxiety
SD: severe depression
SSRI: selective serotonin reuptake inhibitors
STAI: state anxiety subscale of the state-trait anxiety inventory
TNF: Tumor Necrosis Factor
